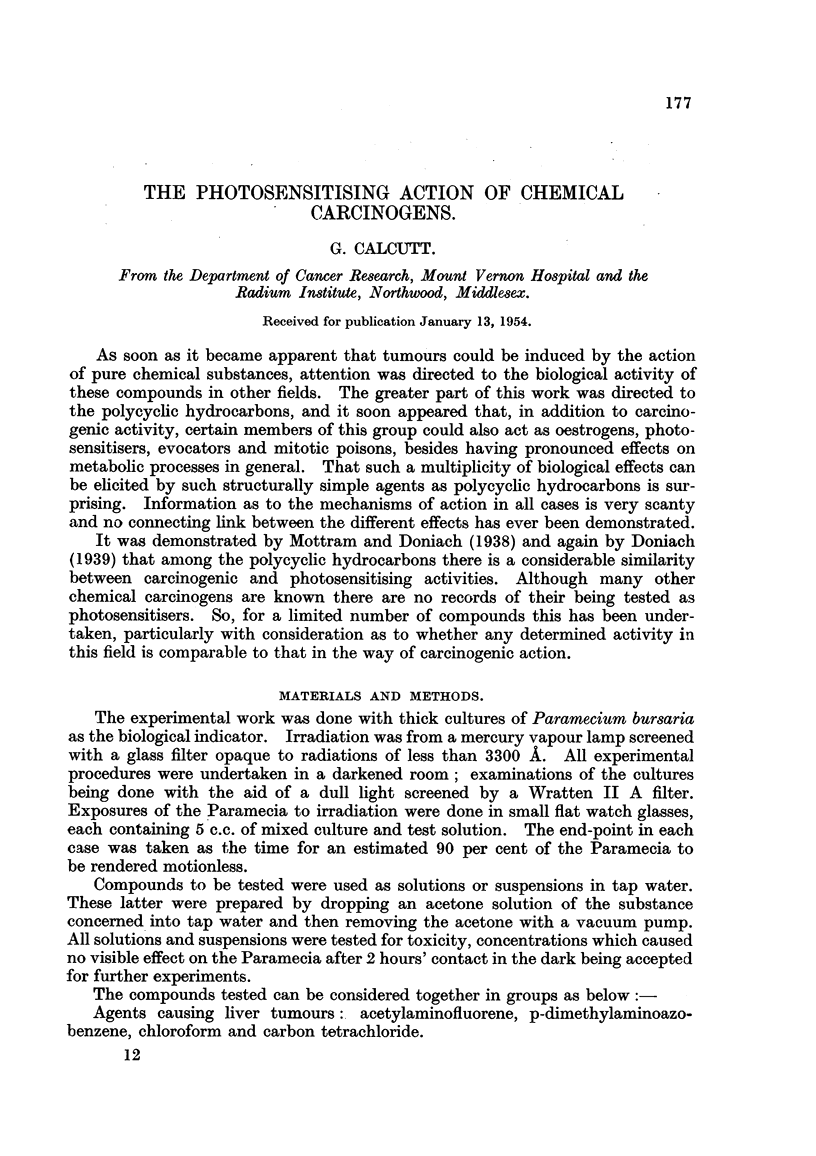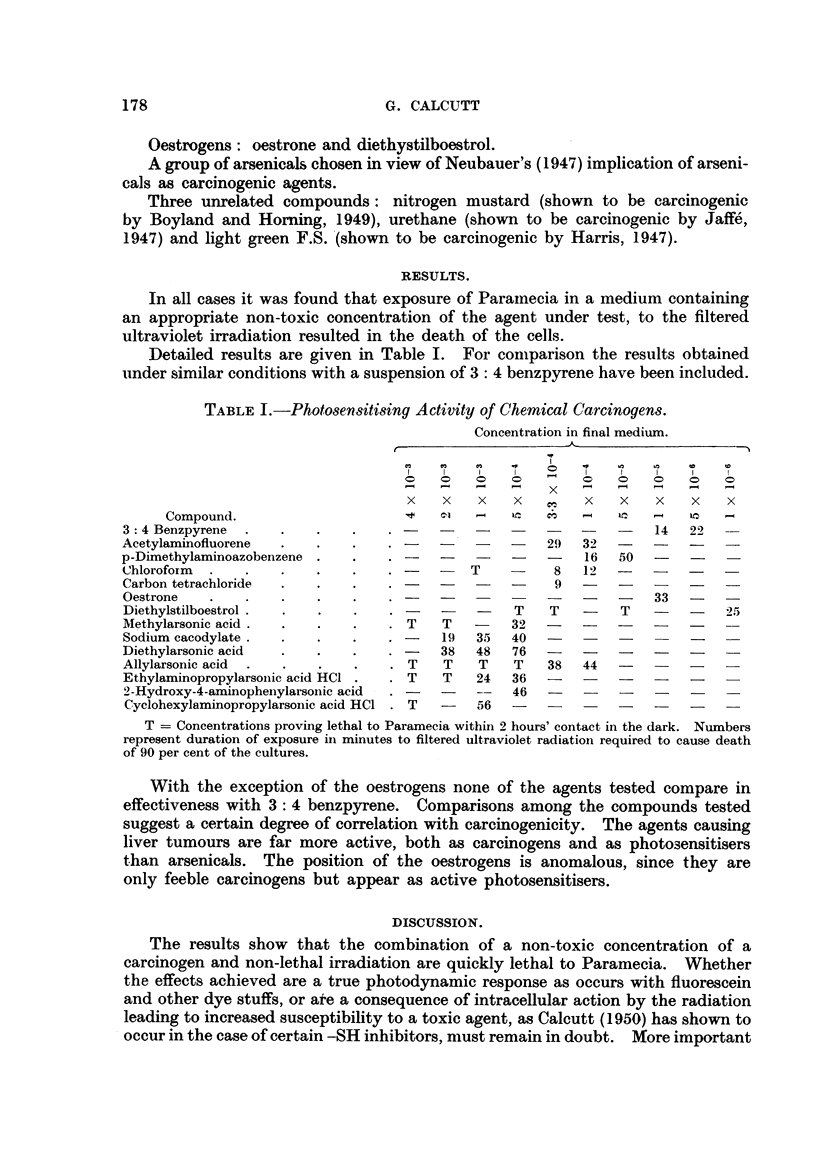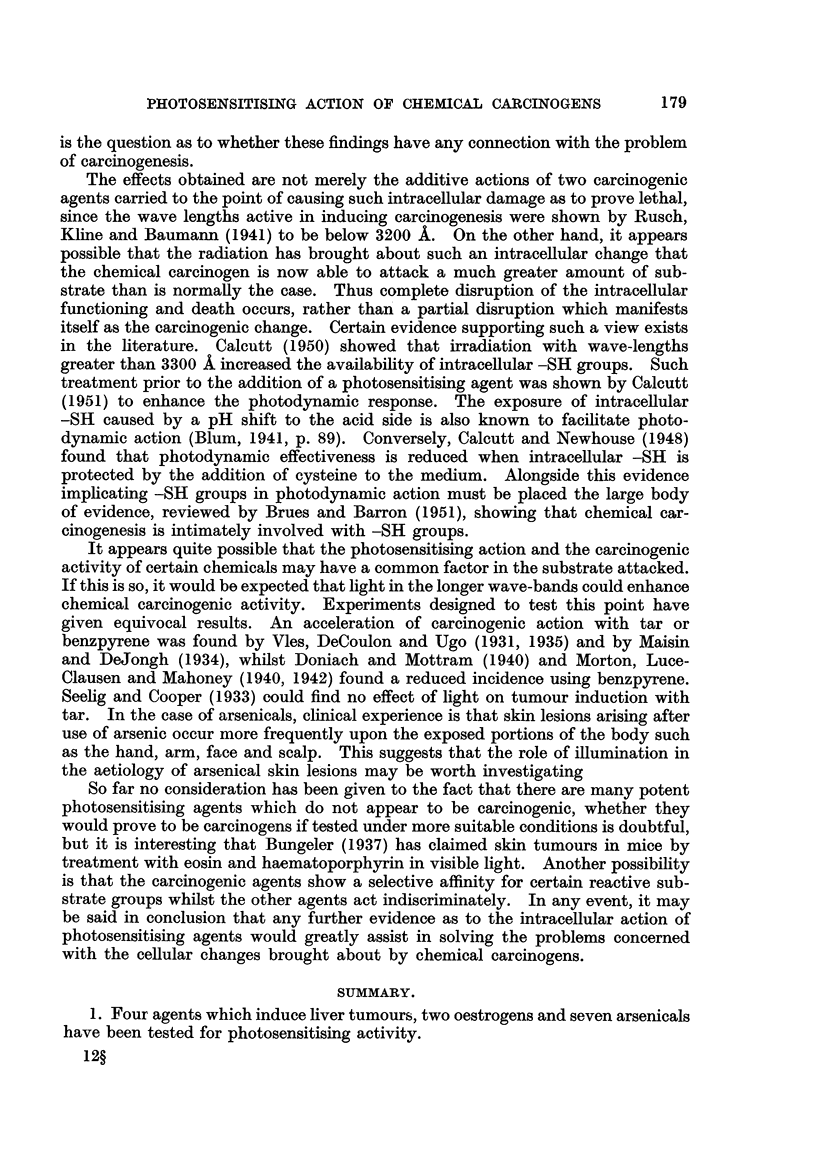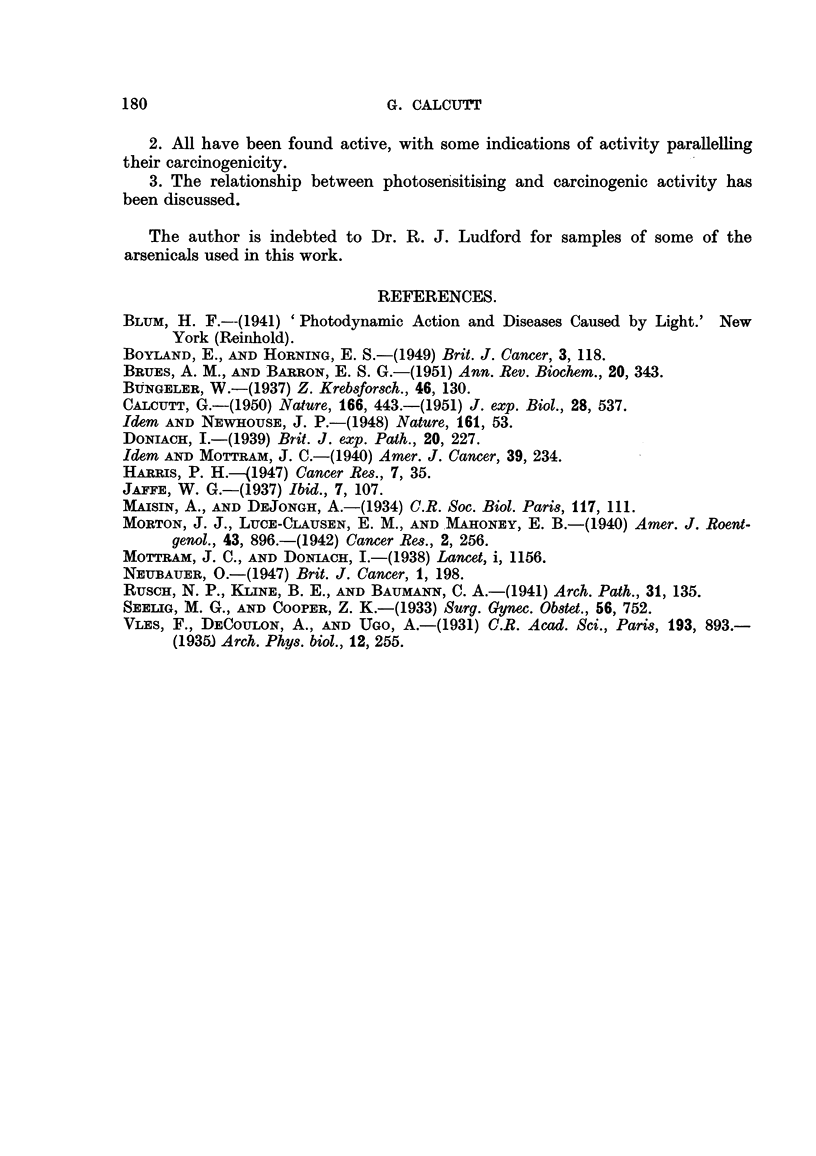# The Photosensitising Action of Chemical Carcinogens

**DOI:** 10.1038/bjc.1954.17

**Published:** 1954-03

**Authors:** G. Calcutt


### THE PHOTOSENSITISING ACTION OF CHEMICAL CARCINOGENS.

G. CALCUTT.

*From the Department of Cancer Research, Mount Vernon Hospital and the Radium Institute, Northwood, Middlesex.*

Received for publication January 13, 1954.

As soon as it became apparent that tumours could be induced by the action of pure chemical substances, attention was directed to the biological activity of these compounds in other fields. The greater part of this work was directed to the polycyclic hydrocarbons, and it soon appeared that, in addition to carcinogenic activity, certain members of this group could also act as oestrogens, photosensitisers, evocators and mitotic poisons, besides having pronounced effects on metabolic processes in general. That such a multiplicity of biological effects can be elicited by such structurally simple agents as polycyclic hydrocarbons is surprising. Information as to the mechanisms of action in all cases is very scanty and no connecting link between the different effects has ever been demonstrated.

It was demonstrated by Mottram and Doniach (1938) and again by Doniach (1939) that among the polycyclic hydrocarbons there is a considerable similarity between carcinogenic and photosensitising activities. Although many other chemical carcinogens are known there are no records of their being tested as photosensitisers. So, for a limited number of compounds this has been undertaken, particularly with consideration as to whether any determined activity in this field is comparable to that in the way of carcinogenic action.

### MATERIALS AND METHODS.

The experimental work was done with thick cultures of *Paramecium bursaria* as the biological indicator. Irradiation was from a mercury vapour lamp screened with a glass filter opaque to radiations of less than 3300 Å. All experimental procedures were undertaken in a darkened room ; examinations of the cultures being done with the aid of a dull light screened by a Wratten II A filter. Exposures of the Paramecia to irradiation were done in small flat watch glasses, each containing 5 c.c. of mixed culture and test solution. The end-point in each case was taken as the time for an estimated 90 per cent of the Paramecia to be rendered motionless.

Compounds to be tested were used as solutions or suspensions in tap water. These latter were prepared by dropping an acetone solution of the substance concerned into tap water and then removing the acetone with a vacuum pump. All solutions and suspensions were tested for toxicity, concentrations which caused no visible effect on the Paramecia after 2 hours' contact in the dark being accepted for further experiments.

The compounds tested can be considered together in groups as below :—

Agents causing liver tumours : acetylaminofluorene, p-dimethylaminoazobenzene, chloroform and carbon tetrachloride.

Oestrogens : oestrone and diethystilboestrol.

A group of arsenicals chosen in view of Neubauer's (1947) implication of arsenicals as carcinogenic agents.

Three unrelated compounds : nitrogen mustard (shown to be carcinogenic by Boyland and Horning, 1949), urethane (shown to be carcinogenic by Jaffé, 1947) and light green F.S. (shown to be carcinogenic by Harris, 1947).

### RESULTS.

In all cases it was found that exposure of Paramecia in a medium containing an appropriate non-toxic concentration of the agent under test, to the filtered ultraviolet irradiation resulted in the death of the cells.

Detailed results are given in Table I. For comparison the results obtained under similar conditions with a suspension of 3 : 4 benzpyrene have been included.

TABLE I.—*Photosensitising Activity of Chemical Carcinogens.*

| | Concentration in final medium. | | | | | | | | | |
|---|---|---|---|---|---|---|---|---|---|---|
| Compound. | $4 \times 10^{-3}$ | $2 \times 10^{-3}$ | $1 \times 10^{-3}$ | $5 \times 10^{-4}$ | $3.3 \times 10^{-4}$ | $1 \times 10^{-4}$ | $5 \times 10^{-5}$ | $1 \times 10^{-5}$ | $5 \times 10^{-6}$ | $1 \times 10^{-6}$ |
| 3 : 4 Benzpyrene | — | — | — | — | — | — | — | 14 | 22 | — |
| Acetylaminofluorene | — | — | — | — | 29 | 32 | — | — | — | — |
| p-Dimethylaminoazobenzene | — | — | — | — | — | 16 | 50 | — | — | — |
| Chloroform | — | — | T | — | 8 | 12 | — | — | — | — |
| Carbon tetrachloride | — | — | — | — | 9 | — | — | — | — | — |
| Oestrone | — | — | — | — | — | — | — | 33 | — | — |
| Diethylstilboestrol | — | — | — | T | T | — | T | — | — | 25 |
| Methylarsonic acid | T | T | — | 32 | — | — | — | — | — | — |
| Sodium cacodylate | — | 19 | 35 | 40 | — | — | — | — | — | — |
| Diethylarsonic acid | — | 38 | 48 | 76 | — | — | — | — | — | — |
| Allylarsonic acid | T | T | T | T | 38 | 44 | — | — | — | — |
| Ethylaminopropylarsonic acid HCl | T | T | 24 | 36 | — | — | — | — | — | — |
| 2-Hydroxy-4-aminophenylarsonic acid | — | — | — | 46 | — | — | — | — | — | — |
| Cyclohexylaminopropylarsonic acid HCl | T | — | 56 | — | — | — | — | — | — | — |

T = Concentrations proving lethal to Paramecia within 2 hours' contact in the dark. Numbers represent duration of exposure in minutes to filtered ultraviolet radiation required to cause death of 90 per cent of the cultures.

With the exception of the oestrogens none of the agents tested compare in effectiveness with 3 : 4 benzpyrene. Comparisons among the compounds tested suggest a certain degree of correlation with carcinogenicity. The agents causing liver tumours are far more active, both as carcinogens and as photosensitisers than arsenicals. The position of the oestrogens is anomalous, since they are only feeble carcinogens but appear as active photosensitisers.

### DISCUSSION.

The results show that the combination of a non-toxic concentration of a carcinogen and non-lethal irradiation are quickly lethal to Paramecia. Whether the effects achieved are a true photodynamic response as occurs with fluorescein and other dye stuffs, or are a consequence of intracellular action by the radiation leading to increased susceptibility to a toxic agent, as Calcutt (1950) has shown to occur in the case of certain –SH inhibitors, must remain in doubt. More important is the question as to whether these findings have any connection with the problem of carcinogenesis.

The effects obtained are not merely the additive actions of two carcinogenic agents carried to the point of causing such intracellular damage as to prove lethal, since the wave lengths active in inducing carcinogenesis were shown by Rusch, Kline and Baumann (1941) to be below 3200 Å. On the other hand, it appears possible that the radiation has brought about such an intracellular change that the chemical carcinogen is now able to attack a much greater amount of substrate than is normally the case. Thus complete disruption of the intracellular functioning and death occurs, rather than a partial disruption which manifests itself as the carcinogenic change. Certain evidence supporting such a view exists in the literature. Calcutt (1950) showed that irradiation with wave-lengths greater than 3300 Å increased the availability of intracellular –SH groups. Such treatment prior to the addition of a photosensitising agent was shown by Calcutt (1951) to enhance the photodynamic response. The exposure of intracellular –SH caused by a pH shift to the acid side is also known to facilitate photodynamic action (Blum, 1941, p. 89). Conversely, Calcutt and Newhouse (1948) found that photodynamic effectiveness is reduced when intracellular –SH is protected by the addition of cysteine to the medium. Alongside this evidence implicating –SH groups in photodynamic action must be placed the large body of evidence, reviewed by Brues and Barron (1951), showing that chemical carcinogenesis is intimately involved with –SH groups.

It appears quite possible that the photosensitising action and the carcinogenic activity of certain chemicals may have a common factor in the substrate attacked. If this is so, it would be expected that light in the longer wave-bands could enhance chemical carcinogenic activity. Experiments designed to test this point have given equivocal results. An acceleration of carcinogenic action with tar or benzpyrene was found by Vles, DeCoulon and Ugo (1931, 1935) and by Maisin and DeJongh (1934), whilst Doniach and Mottram (1940) and Morton, Luce-Clausen and Mahoney (1940, 1942) found a reduced incidence using benzpyrene. Seelig and Cooper (1933) could find no effect of light on tumour induction with tar. In the case of arsenicals, clinical experience is that skin lesions arising after use of arsenic occur more frequently upon the exposed portions of the body such as the hand, arm, face and scalp. This suggests that the role of illumination in the aetiology of arsenical skin lesions may be worth investigating

So far no consideration has been given to the fact that there are many potent photosensitising agents which do not appear to be carcinogenic, whether they would prove to be carcinogens if tested under more suitable conditions is doubtful, but it is interesting that Bungeler (1937) has claimed skin tumours in mice by treatment with eosin and haematoporphyrin in visible light. Another possibility is that the carcinogenic agents show a selective affinity for certain reactive substrate groups whilst the other agents act indiscriminately. In any event, it may be said in conclusion that any further evidence as to the intracellular action of photosensitising agents would greatly assist in solving the problems concerned with the cellular changes brought about by chemical carcinogens.

### SUMMARY.

1. Four agents which induce liver tumours, two oestrogens and seven arsenicals have been tested for photosensitising activity.

2. All have been found active, with some indications of activity parallelling their carcinogenicity.

3. The relationship between photosensitising and carcinogenic activity has been discussed.

The author is indebted to Dr. R. J. Ludford for samples of some of the arsenicals used in this work.

### REFERENCES.

BLUM, H. F.—(1941) 'Photodynamic Action and Diseases Caused by Light.' New York (Reinhold).
BOYLAND, E., AND HORNING, E. S.—(1949) *Brit. J. Cancer*, **3**, 118.
BRUES, A. M., AND BARRON, E. S. G.—(1951) *Ann. Rev. Biochem.*, **20**, 343.
BÜNGELER, W.—(1937) *Z. Krebsforsch.*, **46**, 130.
CALCUTT, G.—(1950) *Nature*, **166**, 443.—(1951) *J. exp. Biol.*, **28**, 537.
*Idem* AND NEWHOUSE, J. P.—(1948) *Nature*, **161**, 53.
DONIACH, I.—(1939) *Brit. J. exp. Path.*, **20**, 227.
*Idem* AND MOTTRAM, J. C.—(1940) *Amer. J. Cancer*, **39**, 234.
HARRIS, P. H.—(1947) *Cancer Res.*, **7**, 35.
JAFFE, W. G.—(1937) *Ibid.*, **7**, 107.
MAISIN, A., AND DEJONGH, A.—(1934) *C.R. Soc. Biol. Paris*, **117**, 111.
MORTON, J. J., LUCE-CLAUSEN, E. M., AND MAHONEY, E. B.—(1940) *Amer. J. Roentgenol.*, **43**, 896.—(1942) *Cancer Res.*, **2**, 256.
MOTTRAM, J. C., AND DONIACH, I.—(1938) *Lancet*, i, 1156.
NEUBAUER, O.—(1947) *Brit. J. Cancer*, **1**, 198.
RUSCH, N. P., KLINE, B. E., AND BAUMANN, C. A.—(1941) *Arch. Path.*, **31**, 135.
SEELIG, M. G., AND COOPER, Z. K.—(1933) *Surg. Gynec. Obstet.*, **56**, 752.
VLES, F., DECOULON, A., AND UGO, A.—(1931) *C.R. Acad. Sci., Paris*, **193**, 893.—(1935) *Arch. Phys. biol.*, **12**, 255.